# Isolation of Anti-Ricin Protective Antibodies Exhibiting High Affinity from Immunized Non-Human Primates

**DOI:** 10.3390/toxins8030064

**Published:** 2016-03-03

**Authors:** Tal Noy-Porat, Ronit Rosenfeld, Naomi Ariel, Eyal Epstein, Ron Alcalay, Anat Zvi, Chanoch Kronman, Arie Ordentlich, Ohad Mazor

**Affiliations:** 1 Department of Biochemistry and Molecular Genetics, Israel Institute for Biological Research, Ness-Ziona 76100, Israel; taln@iibr.gov.il (T.N.-P.); ronitr@iibr.gov.il (R.R.); naomia@iibr.gov.il (N.A.); rona@iibr.gov.il (R.A.); anatz@iibr.gov.il (A.Z.); chanochk@iibr.gov.il (C.K.); arieo@iibr.gov.il (A.O.); 2 Department of Biotechnology, Israel Institute for Biological Research, Ness-Ziona 76100, Israel; eyale@iibr.gov.il

**Keywords:** ricin, antibody, neutralization, affinity, immunization, non-human primates

## Abstract

Ricin, derived from the castor bean plant *Ricinus communis*, is one of the most potent and lethal toxins known, against which there is no available antidote. To date, the use of neutralizing antibodies is the most promising post-exposure treatment for ricin intoxication. The aim of this study was to isolate high affinity anti-ricin antibodies that possess potent toxin-neutralization capabilities. Two non-human primates were immunized with either a ricin-holotoxin- or subunit-based vaccine, to ensure the elicitation of diverse high affinity antibodies. By using a comprehensive set of primers, immune scFv phage-displayed libraries were constructed and panned. A panel of 10 antibodies (five directed against the A subunit of ricin and five against the B subunit) was isolated and reformatted into a full-length chimeric IgG. All of these antibodies were found to neutralize ricin *in vitro*, and several conferred full protection to ricin-intoxicated mice when given six hours after exposure. Six antibodies were found to possess exceptionally high affinity toward the toxin, with *K*_D_ values below pM (*k*_off_ < 1 × 10^−7^ s^−1^) that were well correlated with their ability to neutralize ricin. These antibodies, alone or in combination, could be used for the development of a highly-effective therapeutic preparation for post-exposure treatment of ricin intoxication.

## 1. Introduction

Ricin toxin, derived from the castor bean plant *Ricinus communis*, is a member of the type 2 ribosome-inactivating proteins (RIP) family [1]. The toxin consists of two subunits linked by a disulfide bridge. The ricin B subunit (RTB) is a lectin that binds to galactose residues at the cell surface, allowing the toxin to be internalized by endocytosis and transported to the endoplasmic reticulum, where the disulfide bonds connecting the two subunits are reduced [2,3]. The reduction of the disulfide bonds allows the release of the catalytically-active ricin A subunit (RTA) into the cytoplasm, where it depurinates a conserved adenine residue within a highly conserved stem and loop structure in the 28S ribosomal RNA of the 60S subunit [4]. This process leads to irreversible inhibition of protein synthesis and eventually to cell death [5]. Due to its high toxicity, availability and ease of preparation, ricin was classified as a Category B agent by the U.S. Centers for Disease Control and Prevention (CDC) and is considered a potential bioterror agent. To date, there is no effective treatment against ricin poisoning, and many efforts are dedicated to the development of an efficient therapy. While several studies focus on the development of a vaccine against the toxin [6,7,8], passive immunization remains the most effective post-exposure therapy. Indeed, other studies were conducted aiming to develop anti-ricin antibodies for both prophylaxis and post-exposure treatments [9,10,11,12,13,14,15]. Several anti-ricin antibodies were chimerized [16,17] or underwent humanization [18] while retaining their neutralization capacity. Moreover, it was recently demonstrated that co-administration of doxycycline and ricin-neutralizing antibodies as a post-exposure treatment can improve the survival rate of ricin-intoxicated mice [19]. We have also found that proper immunization with the ricin-holotoxin resulted in the generation of potent polyclonal antibodies that exhibit high affinity and avidity [20].

It was therefore hypothesized that by incorporating immunization methodologies that promote high affinity anti-ricin antibodies *in vivo* in conjunction with efficient screening methods to isolate these antibodies *in vitro*, we could create a panel of potent monoclonal antibodies for treatment against ricin intoxication. In this study, we present the approach taken to reach this end and describe the immunization strategy and the antibody selection procedures employed to ensure the generation and identification of high affinity anti-ricin antibodies.

## 2. Results

### 2.1. Immunization and Characterization of Elicited Antibodies

**Figure 1:**
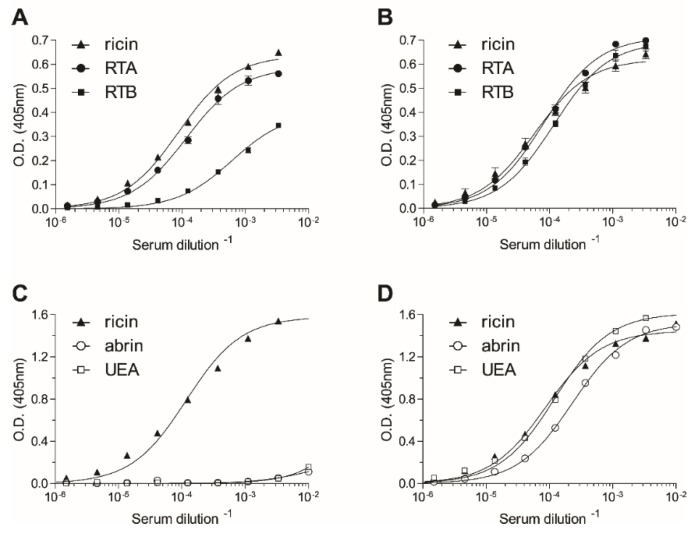
Characterization of the elicited polyclonal anti-ricin antibodies. The reactivity profile of the antibodies elicited by immunization with either ricin holotoxin (**A**,**C**) or with the subunit-based vaccine (**B**,**D**) were determined by enzyme linked immunosorbent assay (ELISA) using the indicated antigens as the coated layer. Points are the mean ± SEM of triplicates (**A**,**B**) or of a representative experiment (**C**,**D**). RTA, ricin A chain; RTB, ricin B chain; UEA, *Ulex europaeus*.

Since a previous study indicated that ricin-holotoxin-based immunization of rabbits induced the production of high affinity antibodies against both subunits of the toxin [20], the same approach was undertaken in the present study for the immunization of non-human primates. To expand the repertoire of elicited anti-ricin antibodies, two rhesus macaques were immunized by different immunization strategies. The first animal was immunized with the holotoxin, while the second was immunized with a subunit-based vaccine consisting of reduced and alkylated holotoxin comprising the two subunits, RTA and RTB [21]. By the end of the immunization course, both animals developed high anti-ricin titers (1:300,000–500,000). To determine whether the two immunization protocols elicited anti-ricin antibodies with different specificities, the relative distribution of antibodies recognizing each of the two subunits of ricin was assessed by enzyme linked immunosorbent assay (ELISA). The half dilution value (Dil_50_) [21] of the serum of the holotoxin-toxin-immunized animal toward the native toxin is 11,400 (Figure 1A). A comparison of the Dil_50_ values for RTA and RTB (8600 and 1600, respectively) established that the antibody response elicited by the holotoxin-toxin immunization was mainly directed toward RTA. In contrast, the animal that was immunized with the subunit-based vaccine developed comparable responses toward RTA and RTB (Dil_50_ of 12,200 and 8600, respectively; Figure 1B).

These results prompted us to further appraise the antibody repertoire elicited by the two immunization strategies. As ricin shares high homology to abrin, a highly toxic plant-derived RIP, we asked whether the immunization protocols induced antibodies that were cross-reactive with the two toxins. Interestingly, antibodies raised toward the holotoxin-based vaccine did not recognize abrin (Figure 1C), while those raised against the subunit vaccine exhibited high cross-reactivity with abrin (Figure 1D). Being plant lectins, both ricin and abrin carry plant-derived sugar moieties that are known to be immunogenic, and it was therefore of interest to determine whether these moieties are the target for these cross-reactive antibodies. To address this question, we performed an ELISA using another plant lectin, *Ulex europaeus* (UEA) [22], which does not share protein-sequence homology with either ricin or abrin, but displays appended glycans, whose composition and structure are the same as ricin and abrin. Indeed, the antibodies raised by the subunit-based vaccine reacted very efficiently with UEA (Figure 1D), suggesting that the major antibody response was elicited toward the sugar moieties of the toxin (no anti-sugar antibodies could be found prior to the immunization process). It should be noted that while this anti-serum can efficiently neutralize ricin *in vitro*, it does not neutralize abrin (data not shown), indicating that it also contains a small, yet substantial set of ricin-specific neutralizing antibodies. Taken together, the results presented so far indicate that the applied immunization protocols elicited a diverse antibody response, which can serve as a high-quality source for the isolation of a wide spectrum of monoclonal antibodies.

### 2.2. Isolation of Anti-Ricin Antibodies

Panning of single-chain Fv (scFv) phage display libraries, based on antibody-encoding genes isolated from the lymphatic organs of immunized animals (“immunized libraries”), was proven to be a highly efficient tool for the isolation of high affinity antibodies [23,24]. Utilization of a wide panel of primers that provide extensive coverage of the VH/VL-encoding genes is mandatory for successful isolation of high-quality antibodies from immunized libraries. Based on data mining, which included a literature search of the published primers used to amplify non-human primate VH/VL genes, as well as reviewing gene bank databases [25,26], a set of 136 primer pairs, which provide improved coverage of all known rhesus VH, Vκ and Vλ germline families, was constructed (Figure S1) and used to amplify VH- and VL-encoding mRNA from the lymphatic organs and peripheral blood of the ricin-immunized animals. The VH and Vκ/Vλ gene pool of each animal was assembled by PCR to obtain combinatorial scFv fragments that were inserted into a phagemid vector to create two separate libraries that were subjected to three rounds of panning against biotin-labeled ricin. Since the antibody repertoire displayed on the phage surface probably reflects that of the circulatory antibodies, it was hypothesized that in the case of the library originating from the subunit-based vaccine animal, most of the panned anti-ricin antibodies would target the sugar moieties and therefore would not be useful for toxin neutralization. To circumvent this possible outcome, the third round of panning of this library was performed in the presence of an excess of UEA to compete with ricin for the binding of the anti-sugar antibodies. Indeed, the inclusion of UEA completely abolished the binding of anti-sugar antibodies to ricin (93% of the ricin-binding clones at the onset of the third panning were directed against the sugar moieties). By the end of the panning process, individual clones were screened by phage-ELISA for their ability to bind ricin. Upon fingerprinting analysis and sequencing of the genes encoding ricin-specific binding clones, 10 clones were selected (Table 1). Seven of these clones originated from the subunit-based vaccine library, of which antibody MH1 accounted for 65% of the positive clones and antibodies MH2 and MH75 accounted for 20%. Three antibodies were chosen from the holotoxin-based vaccine library, of which antibody MH36 accounted for 50% of the positive clones.

**Figure 2:**
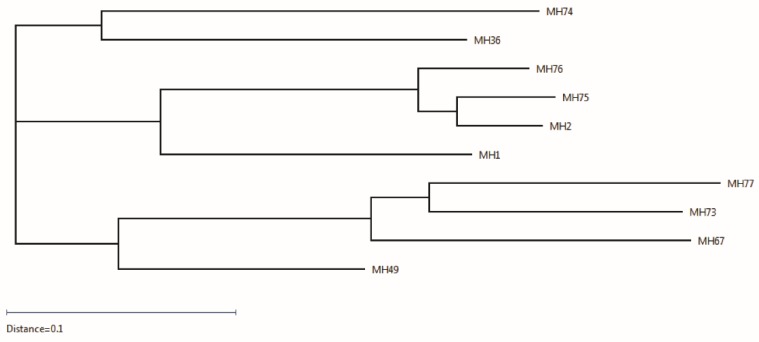
Grouping of the 10 scFvs in the form of a phylogenetic tree. The scFvs are grouped into three main clusters.

Sequences of the 10 selected clones were analyzed and compared to the Rhesus germline V, D and J genes using IgBlast, and the most similar Rhesus germline genes were assigned to each clone (Table 2). Additionally, all sequences were analyzed by multiple alignments (Figure S2), enabling the generation of a phylogenetic tree that reflects the divergence between the different sequences (Figure 2). This analysis revealed that antibodies MH2 and MH75 originated from one germline recombination (Table 2) and the differences in their sequences probably stem from the somatic hypermutation process. Antibody MH76 also clusters with these two antibodies, since it shares the same V-germline gene as these two antibodies (yet, with different D–J genes). The other antibodies isolated from the same library originated from other diverse germline genes′ recombinations, as indicated by their clustering in the phylogenetic tree (Figure 2). In total, five IGHV genes were combined with seven IGHD genes and five IGHJ genes. The seven λ light chains were coded by four IGLV genes and three IGLJ genes, while the three *κ* light chain clones were coded by three IGKV genes combined with the same IGKJ gene (Table 2). In accordance with previous reports [28,29,30,31], the IGHV genes originated mostly from two subgroups (IGHV3 and 4), while the IGHD and the IGHJ genes were more diverse. Interestingly, two of the antibodies isolated from the holotoxin-based vaccine library share the same VDJ-germline genes of the VH, with different VL genes, probably due to the combinatorial assembly of the same VH chain with two different VL chains during library generation. Therefore, the phylogenetic tree mostly reflects similarities in the light chain, dividing the 10 sequences into three clusters: one cluster consisting of the three clones having a *κ* light chain (MH67, MH73, MH77) together with MH49, which shares the same VH as MH67, and the other two clusters consisting of clones with λ light chain and divided according to their IGLV gene subgroup, with MH36 and MH74 having genes belonging to the IGLV1 subgroup and the others to the IGLV3 subgroup. It should be noted that these two subgroups were previously suggested by Sundling *et al.* to be frequently used in rhesus macaques [31].

### 2.3. Epitope Binning

To enable further characterization, the isolated antibodies were reformatted and expressed as chimeric, human-like antibodies [23] composed of macaque variable chains and human constant regions (IgG1/*κ*/λ according to the original sub-typing of each clone). To determine the ricin subunit specificity of the antibodies, ELISA was performed using isolated ricin subunits as the coated antigen. All three antibodies derived from the holotoxin-based vaccine library were found to target the RTA subunit, while only two of the seven antibodies from the reduced subunit-based library recognized RTA, all others targeting RTB (Table 1). Interestingly, antibody MH73 recognized neither RTA nor RTB by ELISA, probably since its target epitope is not well exposed in the solid-phase bound format. MH73 binding to soluble subunits was therefore studied using the Octet Red biolayer interferometry system. Biotinylated MH73 was immobilized to the Octet sensor, and the binding profile to each ricin subunit was monitored, revealing that it binds RTB (Figure S3). These results are in good agreement with our aforementioned findings that the majority of the elicited polyclonal antibodies by the holotoxin-based immunization are against RTA, while the subunit-based vaccine induced antibodies against both subunits.

**Figure 3:**
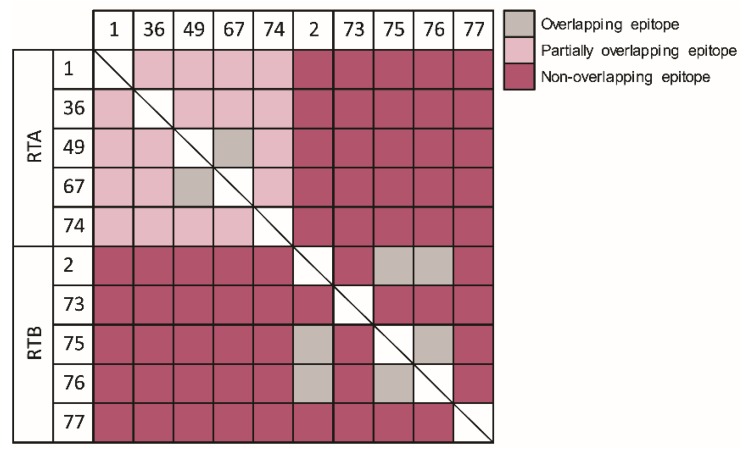
Epitope binning. The ability of each antibody to bind simultaneously with each of the other antibodies was tested using the Octet Red system. Following immobilization of the first antibody to the sensor, ricin was loaded, and the ability of the next antibody to bind the pre-formed complex was assessed.

Next, we sought to cluster the antibody panel according to their binding site. To this end, epitope binning was performed using Octet Red. Each individual antibody was biotinylated, immobilized to the sensor, loaded with ricin and then incubated with each of the other antibodies. Simultaneous binding of the second antibody to ricin induces a wavelength shift in the interference pattern, which indicates that the two antibodies bind to non-overlapping epitopes [32]. Conversely, if the two antibodies bind the same or partially-overlapping epitope on ricin, no or a low wavelength shift, respectively, is induced. Although the anti-RTA antibodies were isolated from two animals, immunized by two different vaccines, we could determine that the entire set of antibodies directed toward RTA binds to partially-overlapping epitopes, with the exception of MH49 and MH67, which bind the same epitope (Figure 3). On the other hand, three distinct non-overlapping binding sites were found on RTB, and not-surprisingly, MH2, MH75 and MH76 (that originated from the same antibody V-germline gene) bind the same epitope. Each of the anti-RTA antibodies could bind simultaneously with all of the antibodies directed against RTB (and *vice versa*).

### 2.4. In Vitro Neutralization of Ricin Cytotoxicity

We have recently developed a novel *in vitro* assay to assess the neutralization potency of anti-ricin antibodies [27], and as a proof of concept, we demonstrated, using antibodies MH1, MH74 and MH75, that monoclonal antibodies could be evaluated in this assay. Here, we determined the neutralizing potency of the other antibodies that were isolated from the immunized libraries. To this end, ricin (30 ng/mL) was incubated with increasing concentrations of the chimeric antibodies, and the mixtures were added to Ub-FL cells. Seven hours later, the residual intracellular luciferase levels were measured, and the antibody concentration needed to neutralize 50% of the ricin activity (ED_50_) was determined. It was found that the ED_50_ values are between 500 and 52,000 ng/mL (Table 1), the most potent antibodies neutralizing ricin at about a 3–10-fold molar excess (the ricin concentration was 0.5 nM, and the antibodies′ concentration at the ED_50_ point were about 5 nM). Interestingly, even though the entire panel of anti-ricin antibodies was isolated merely based on their ability to bind ricin, they all have the capability to neutralize the toxin.

### 2.5. Affinity of the Anti-Ricin Antibodies

**Figure 4:**
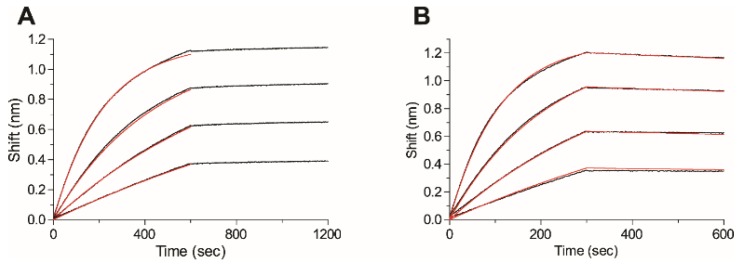
Affinity measurements. The affinity of antibody (**A**) MH1 or (**B**) MH2 was measured using bio-layer interferometry. Biotinylated antibodies were immobilized on a streptavidin-biosensor and reacted for 300–500 s with increasing concentrations of ricin (black lines; from bottom up: 6.25 nM, 12.5 nM, 25 nM and 50 nM). The sensors were then immersed in buffer for another 300–600 s (dissociation phase). Red lines: curve fitting of the 1:1 binding model. The dissociation phase of MH1 (even if monitored for several hours) could not be fitted, since the off rates are below the detection limit (1 × 10^−7^ s^−1^).

Our next goal was to measure the affinity of the anti-ricin panel of antibodies toward ricin using the Octet Red biolayer interferometry system. Each antibody was biotinylated, immobilized on the Octet sensor and monitored for its ricin binding profile (at different concentrations). The sensorgrams were fitted with a 1:1 binding model, and the association (*k*_on_) and the dissociation (*k*_off_) constants were determined (Figure 4 depicts the binding profiles of antibodies MH1 and MH2 as representative samples). The *k*_on_ values were in the same range for the entire antibody panel (7–35 × 10^4^ Ms^−1^; Table 1). However, to our great surprise, huge variations in *k*_off_ values were observed. The dissociation constant of four antibodies (MH2, MH67, MH74 and MH76) ranged from 0.7–10 × 10^−4^ s^−1^ (Figure 1B); hence, they could be easily fitted, enabling us to determine their affinity toward ricin (*K*_D_ of 0.6–45 nM). However, the dissociation rate of the additional antibodies was extremely slow (below the Octet Red detection limit, 1 × 10^−7^ s^−1^) and could not be measured (MH1 in Figure 1A as a representative of this group) even when the dissociation phase was monitored for several hours, indicating that their affinity is in the sub-pM range. The only exception was antibody MH77, whose dissociation constant was close to the detection limit, thus exhibiting a *K*_D_ value of 1 pM (Table 1). Additional confirmation for the extremely tight binding between ricin and antibodies MH1, MH36, MH49, MH73 and MH75 was obtained from the fact that even upon exposing the antigen-antibodies complexes to a highly acidic environment (pH 2.7), no dissociation could be observed. Even though the exact affinity values of the tight-binder antibodies could not be fully determined, to the best of our knowledge, this is the first report of anti-ricin antibodies that exhibit such high affinities. Attempts will be made in the future to evaluate their dissociation constants by other biophysical approaches.

### 2.6. Post-Exposure Treatment of Ricin Intoxication

**Figure 5:**
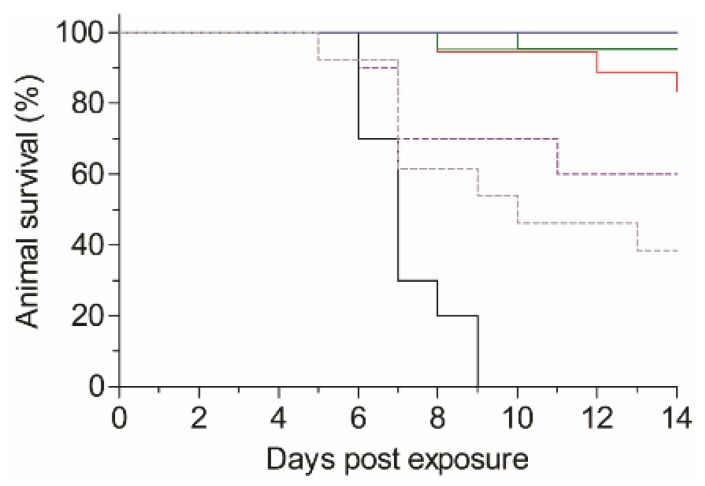
Post-exposure treatment of ricin-intoxicated mice. Mice were intranasally instilled with ricin (5 µg/kg; 2LD_50_). Six hours later, the ricin-intoxicated mice were intravenously treated with 100 µg of each antibody (*n* = 10–22), and animal survival was monitored for 14 days. Black line: untreated, ricin-intoxicated mice. Blue line: MH1/MH77. Green line: MH36/MH75. Red line: MH73. Dashed purple line: MH76. Dashed gray line: MH2.

The results presented so far, indicating that the isolated antibodies are of extremely high affinity and capable of neutralizing ricin *in vitro*, prompted us to evaluate their ability to confer post-exposure protection to ricin-intoxicated mice. In this model, mice were exposed to pulmonary intoxication of 2LD_50_ of ricin succumb within 5–9 days (mean time to death of 6.5 days; Figure 5). To assess the activity of the antibodies, we selected MH1 and MH36 as representatives of the anti-RTA group and MH73, MH75 and MH77 for the anti-RTB in the assay. We also included antibodies MH2 and MH76, which bind the same epitope as MH75, but display lower affinity toward ricin. At six hours post-intoxication, mice were treated by intravenous administration of 100 µg of the tested antibodies and were monitored for 14 days. It was found that treatment with MH1, MH36, MH75 or MH77 conferred extremely high animal survival rates of 95%–100% (Figure 5). In the group that was treated with antibody MH73, 85% of the intoxicated mice survived, while in those treated with antibodies MH2 or MH76, a survival rate of 40% and 60%, respectively, was observed.

## 3. Discussion

To date, the use of neutralizing antibodies is the most promising post-exposure treatment for ricin intoxication. Therefore, there is a great interest in identifying efficient antibodies against this lethal toxin. In this study, following immunization of two non-human primates with ricin, a panel of high affinity, highly neutralizing monoclonal antibodies was isolated. Moreover, several of these antibodies were shown to confer full protection to ricin-intoxicated mice when administered six hours post-exposure.

Most studies aiming at developing anti-ricin antibodies have used either the holotoxin or isolated ricin subunits [9,10,12,14,33,34]. Here, to increase the potential antibody repertoire, two different toxin preparations, holotoxin or a mixture of monomeric ricin subunits, were used for immunization. Indeed, a clear difference was observed in the spectrum of antibodies developed following vaccination by the two. Immunization using the holotoxin resulted in highly specific antibodies directed mainly against RTA, while subunit-based immunization elicited an antibody response against both subunits. We have previously demonstrated that immunization of rabbits with a ricin-toxoid [20] elicited an antibody response whose subunit specificity is similar to that induced here by the subunit-based immunization. It is therefore possible that upon immunization with the native holotoxin, ricin interacts differently with the antigen-presenting cells and/or B-cells, in a way that only a limited epitope domain in RTA is available to elicit antibody response. If indeed this is the case, then this issue should be taken into consideration in the future when designing the next generation ricin-based vaccine, and the use of the subunit- or toxoid-based vaccination should be re-considered.

**Figure 6:**
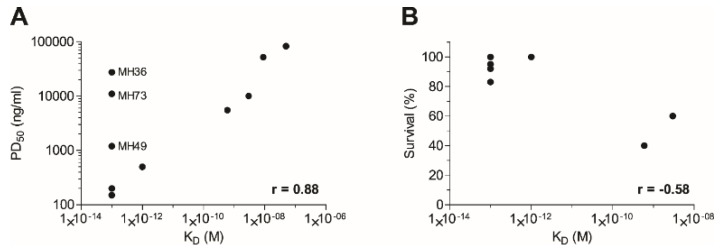
Correlation between antibody affinity and neutralization potency (**A**) *in vitro* or (**B**) *in vivo* when given six hours post-exposure. Compiled from data in Table 1 and Figure 5.

In a recent study, Pincus *et al.* have demonstrated that the murine anti-ricin monoclonal antibody, RAC18, outperformed its chimeric counterpart that was unable to confer good protection when given 4 h post-exposure, in a murine model [17]. Similarly, the chimeric form of antibody GD12 was shown to be less potent *in vivo* than its murine origin antibody [16]. In another study, the chimeric anti-ricin antibody, *c*-PB10, conferred high protection rates in a murine model only when given up to 5 h post-intoxication [35]. These studies attributed these discrepancies to the fact that a non-heterologous Fc was used, thus affecting the pharmacokinetic parameters of the administered ricin-neutralizing antibodies. Here, we found that although human Fc-based antibodies were administrated, intoxicated mice that were treated at six hours post-exposure were fully protected. Moreover, these antibodies provided equivalent (if not better) post-exposure protective efficacy when compared to a rabbit-derived anti-ricin hyperimmune serum that was tested in the same murine intoxication model [19]. It is logical to assume that in a fast-progressing disease, such as ricinosis, a favorable neutralizing-antibody pharmacokinetics behavior will provide a better outcome. We have previously found that antibodies and Fc-fusion protein that share the same human Fc-region as the anti-ricin antibodies exhibit a circulatory half-life of 2–3 days in murine and rabbit models [36,37]. Yet, our data suggest that the exceptional high-affinity characteristics, as found for several of the antibodies, may enable a rapid and potent interaction with the toxin, thus overcoming the fast clearance rate limitation. In fact, the correlation between the affinity of anti-ricin antibodies and neutralization potency was not yet fully addressed. Several studies have suggested that epitope specificity, rather than antibody affinity or isotype, is the primary determinant of the antibody neutralization capacity [7,12,33,34,38]. In contrast, Hu *et al.* have shown that antibody D9, which exhibited the highest affinity towards ricin, was found to provide better neutralization when compared to other anti-ricin antibodies that exhibited lower affinity values [18,39]. By compiling the data gathered in this study (Table 1), a very good correlation (*r* = 0.88, *p* < 0.001) between antibody affinity and the ability to neutralize ricin *in vitro* was found (Figure 6A). Moreover, it is demonstrated, for the first time, that post-exposure protection also correlates (*r* = −0.58) with antibody affinity (Figure 6B). Antibodies MH2, MH75 and MH76 bind the same epitope, as determined by epitope binning experiments (Figure 3), but their neutralization potency differs greatly in a manner correlating with their affinity values (Figure 6). The same is true for MH49 and MH67. Interestingly, antibodies MH36, MH49 and MH73 do not fall within the correlation trend in the *in vitro* assay. Antibodies MH36 and MH49 were found to bind to the same epitope region as MH1 (which possesses the same affinity as these two), yet their neutralization potency is markedly lower. Antibody MH73, which binds to RTB and possesses high affinity towards the toxin, also does not exhibit high neutralization potency *in vitro*. However, when tested *in vivo* for post-exposure protection, both MH36 and MH73 provided very high protection rates, as would be expected based on their affinity values. It could be therefore hypothesized that their binding to the toxin *in vivo* inhibits other mechanisms of toxicity that could not be fully appreciated in the *in vitro* system, such as interactions with other host cells (possibly immune cells). As ricin is a multi-faceted protein that participates in many protein-protein interactions during the intoxication process, we hope in the future to identify the target epitopes of these antibodies and, thus, broaden our understanding of this process.

To conclude, we have isolated a panel of highly potent antibodies against ricin that confer full protection against ricin-intoxication in a murine model when given six hours post-exposure. These antibodies exhibit exceedingly high affinities towards the toxin, which correlate well with their ability to neutralize ricin. The data presented in this study promote the use of these antibodies, as a stand-alone or as a cocktail, as highly effective therapeutic preparations for post-exposure treatment of ricin intoxication.

## 4. Experimental Section

### 4.1. Animal Immunization

Pure ricin was prepared as described previously [19]. Ricin was reduced in the presence of Dithiothreitol (DTT) at pH 9.0 followed by the addition of iodoacetamide and dialysis in Phosphate buffered saline (PBS). Two non-human female primates (*Macaca mulatta*) were immunized with either the subunit-based vaccine, using reduced ricin, or with the native ricin holotoxin. The first animal was injected with 100 µg of reduced ricin (~20 µg/kg), mixed with complete Freund′s adjuvant followed by two monthly booster injections of 100 µg reduced ricin mixed with incomplete Freund′s adjuvant. The second animal was injected with 2 µg of purified ricin mixed with complete Freund′s adjuvant, followed by three monthly booster injections of 5, 80 and 80 µg purified ricin mixed with incomplete Freund′s adjuvant. Seven days after the last boost, the macaques were sacrificed, and samples were taken from their blood and lymphatic nodes.

### 4.2. Primers Design

A set of degenerate primers was designed to amplify all known sequences of macaque immunoglobulin families [25]. A total of 52 forward and reverse primers were designed, giving 136 different primer pairs to amplify macaque VH, Vκ and Vλ sequences. Primer sequences are shown in Figure S1, marked by a dashed line. A second set of primers was designed, consisting of the same gene specific sequences with additional sequences for restriction and cloning (full sequences in Figure S1).

### 4.3. scFv Library Construction

RNA was extracted from lymph nodes using the RNeasy mini kit and from blood samples using the RNeasy Protect Animal Blood kit (Qiagen GmbH, Hilden, Germany) according to the manufacturer′s instruction. All RNA samples were mixed together, and first-strand synthesis was performed using the Verso cDNA synthesis kit (Thermo Scientific, Waltham, MA, USA), with random hexamers and 1 µg RNA. The specific primers designed (marked by a dashed line in Figure S1) were utilized for the amplification of VH, Vκ and Vλ fragments, for each animal separately (1 min 95 °C; 30 cycles of: 20 s, 94 °C; 30 s, 55 °C; 1 min, 72 °C; 5 min, 72 °C) using Advantage 2 DNA polymerase (Clontech, Mountain View, CA, USA). PCR products were purified by 2% agarose gel electrophoresis using the QIAquick Gel extraction kit (Qiagen GmbH, Hilden, Germany), and 100 ng of the first PCR products were used for a second amplification (1 min 95 °C; 20 cycles of: 20 s, 94 °C; 30 s, 57 °C; 1 min, 72 °C; 5 min, 72 °C) with the full primers depicted in Figure S1, using the same reaction mixture, in order to add restriction sites for cloning. The VH, Vκ and Vλ PCR products were then pooled, separately, and the light-chain fragments, Vκ and Vλ, were used as a template for another PCR, under the same conditions, where a linker sequence encoding GGGSx3 was added at the 5′ end of the sequences.

The construction of the scFv library was performed by assembly PCR. The VH and Vκ/Vλ fragments were mixed in a 1:1 ratio together with Taq Polymerase (MyTaq, Bioline, London, UK) and its corresponding reaction mixture (containing buffer, 5 mM dNTPs and 15 mM MgCl_2_) without the addition of primers, for 10 PCR cycles (95 °C, 1 min; 10 cycles of: 95 °C, 15 s; 60 °C, 30 s; 72 °C, 30 s; 72 °C, 5 min). Second, in order to maintain the diversity of the library, the first PCR output was divided into 10 new PCR tubes and fresh reaction mixture was added, including primers designed to assemble both variable regions (VH and Vκ/λ; primer ASS1; Figure S1). Amplification was performed for an additional 30 cycles (95 °C, 1 min; 30 cycles of: 95 °C, 15 s; 65 °C, 20 s; 72 °C, 1 min; 72 °C, 5 min). PCR products were resolved on 1% agarose gel, purified using the QIAquick Gel extraction kit (Qiagen GmbH, Hilden, Germany), and then ligated to the pCC_16_ plasmid [40]. Plasmid and scFvs were digested using *Nco*I*/Not*I restriction enzymes (FastDigest, Thermo Scientific, Waltham, MA, USA) 5 U each, in a 50-µL reaction volume, overnight at 37 °C. The digest was inactivated by heating at 80 °C for 10 min. The vector was purified from 1% agarose gel using the QIAquick Gel extraction kit, and the inserts were purified using the QIAquick PCR purification kit (Qiagen GmbH, Hilden, Germany). The scFv was than cloned into 200 ng of the pCC_16_ vector (5:1 ratio; insert: vector), in a 20-µL reaction volume, using 6 U of ligase (T4 HC ligase, Thermo Scientific, Waltham, MA, USA), with overnight incubation at 4 °C, followed by inactivation at 70 °C for 5 min. Each ligation reaction was transformed to 100 µL of *E. coli* MC1061F′ electrocompetent cells (Lucigen, Middleton, WI, USA), and a total of 20 cloning reactions were performed for each library. The transformed bacteria, containing the final scFv libraries, were plated on YPD agar (BD, Franklin Lakes, NJ, USA) supplemented with 100 µg/mL ampicillin and 100 mM glucose and, after an overnight culture at 30 °C, were harvested, aliquoted and stored at −80 °C.

### 4.4. Library Screening

For library packaging, 200 mL YPD medium, containing 100 µg/mL ampicillin and 100 mM glucose, were inoculated with 0.5 mL of the scFv library. Bacteria were grown in a shaker incubator (New Brunswick Scientific, Enfield, CT, USA) at 37 °C, 220 rpm to an O.D._600_ of 0.7–0.9. Twenty-five milliliters of bacteria were than infected with 125 µL of M13KO7 helper phage (New England Biolabs, Ipswich, MA, USA) by incubating at 37 °C for 30 min without shaking, followed by 30 min at 120 rpm. Infected cells were harvested (5 min, 4000 rpm) and resuspended in 100 mL YPD with 100 µg/mL ampicillin and 50 µg/mL kanamycin. After an overnight culture at 30 °C at 200 rpm, cells were pelleted by centrifugation for 10 min, 4000 rpm at 4 °C, and the supernatant containing the phages was filtered through a 0.45-µm filter and then precipitated with 1/5 volume of 20% PEG6000 (polyethylene glycol)/2.5 M NaCl solution for 2 h on ice. The phages were pelleted by centrifugation for 1 h at 9000× *g*, 4 °C, and resuspended in 5 mL PBS.

For library panning, 100 µL streptavidin-coated magnetic beads (Dynabeads M-280, 10 mg/mL, Invitrogen, Carlsbad, CA, USA) were incubated with 300 µg/mL biotin-labeled ricin (biotinylated using a commercial kit; EZ-Link sulfo-NHS-biotin, Pierce-Thermo Scientific, Waltham, MA, USA) for 30 min with gentle shaking. The ricin solution was then removed, and the beads were blocked with blocking solution (3% BSA in PBS). Approximately 1 × 10^11^ phage clones were blocked with blocking solution for 1 h and then incubated for an additional 90 min with the Dynabeads. Next, the beads were washed 5 times with blocking solution, 6 times with PBST (PBS, 0.05% Tween 20) and twice with PBS, and the beads with the bounded phages were used directly to infect 5 mL of *E. coli* TG1 strain (Lucigen, Middleton, WI, USA) at 37 °C for 30 min without shaking followed by 30 min at 120 rpm. The bacteria were plated on YPD agar plates with 100 µg/mL ampicillin and 100 mM glucose, cultured overnight at 30 °C, and clones were then harvested into 30 mL YPD-100 µg/mL ampicillin-100 mM glucose-20% glycerol solution; 100 µL were used for phage packaging in 25 mL medium, as described above. Two additional panning rounds were conducted as described, except that 10^9^ phage clones were used as input, 50 µL UEA (1 mg/mL) were added to the incubation with the phages and the washing steps included 5 washes with blocking solution, 10 washes with PBST and 2 with PBS. Single colonies were randomly picked from the third panning output, and phages were rescued and tested for their binding to ricin.

### 4.5. ELISA

Maxisorp 96-well microtiter plates (Nunc, Sigma-Aldrich, St. Louis, MO, USA) were coated overnight with 2 µg/mL of ricin, RTA, RTB, abrin [41] or UEA (50 µL/well) in NaHCO_3_ buffer (50 mM, pH 9.6), then washed and blocked with PBST buffer (0.05% Tween 20, 2% BSA in PBS) for one hour. Individual clones or antibodies were added to the plates for a one-hour incubation; the plates were then washed with PBST and incubated with the detecting antibody: horseradish peroxidase (HRP)-conjugated anti-M13 antibody (GE healthcare, Little Chalfont, UK) followed by detection with 3,3′,5,5′-tetramethybenzidine (TMB/E, Millipore, Billerica, MA, USA), for phage clones, or anti-human IgG conjugated to alkaline phosphatase (sigma), for full antibodies. For serum ELISA, plates were coated with 5 µg/mL of either abrin [27] or UEA (Sigma).

### 4.6. Fingerprint Analysis

Following colony PCR, 5 µL of the PCR products were taken directly for restriction with 0.5 µL *Mva*I (FastDigest; Thermo Scientific, Waltham, MA, USA) and 1 µL buffer ×10 (provided by the manufacturer) in a 10-µL reaction volume. Restriction was conducted for one hour at 37 °C, and the entire reaction mix was then resolved on 2% agarose gel.

### 4.7. Nucleic Acid Analysis

Phagemid DNA was isolated using the QIAprep spin Miniprep kit (Qiagen, GmbH, Hilden, Germany), and scFvs were sequenced by the ABI Prism 310 Genetic Analyzer (Applied Biosystems, Foster City, CA, USA) using primers TAB-RI and CBD-AS (Figure S1). Nucleic acids sequences of the VH and VL fragments were compared to the rhesus germline immunoglobulin genes by using the IgBlast tool (http://www.ncbi.nlm.nih.gov/igblast/) [26]. The amino acid sequences corresponding to the 10 scFv sequences were further aligned and displayed in the form of a phylogenetic tree by using the MegAlign Pro tool (Lasergene 11 Core Suit, DNASTAR, Madison, WI, USA, 2013). The total length of the branches connecting any two sequences is in proportion with the differences between these sequences.

### 4.8. Producing Full-Length Antibodies

Phagemid DNA of the desired clones was isolated using QIAprep spin Miniprep kit (Qiagen, GmbH, Hilden, Germany), and VH and VL sequences were cloned into a mammalian full-length immunoglobulin expression vector [42], providing each chain with the corresponding human constant genes and resulting in IgG1/*κ*/λ chimeric macaque-human antibody expression. FreeStyle Max 293 cells (Thermo Scientific, Waltham, MA, USA) were transiently transfected with the vector, and after a week, the supernatant was collected and the antibodies were purified on a HiTrap Protein-A column (GE healthcare, Little Chalfont, UK).

### 4.9. Affinity Measurements and Epitope Binning

Binding studies were carried out using the Octet Red system (ForteBio, Version 8.1, Menlo Park, CA, USA, 2015) that measures biolayer interferometry (BLI). All steps were performed at 30 °C with shaking at 1500 rpm in a black 96-well plate containing 200 µL solution in each well. Streptavidin-coated biosensors were loaded with biotinylated antibody (5 µg/mL) for 300 s followed by a wash. The sensors were then reacted for 300 s with increasing concentrations of ricin and then moved to buffer-containing wells for another 300 s or up to several hours (dissociation phase). Binding and dissociation were measured as changes over time in light interference after subtraction of parallel measurements from unloaded biosensors. Sensorgrams were fitted with a 1:1 binding model using the Octet data analysis software 8.1 (Fortebio, Menlo Park, CA, USA, 2015), and the presented values are an average of several repeated measurements. For the binning experiments of antibodies pairs, antibody-loaded sensors were incubated with a fixed ricin concentration, washed and incubated with the non-labeled antibody pair.

### 4.10. In Vitro Ricin Neutralization Assay

HeLa Ub-FL cells [43] were a kind gift from Professor Piwnica-worms (University of Texas, MD Anderson Cancer Center, Austin, TX, USA). Cells were cultured in Dulbecco′s Modified Eagle′s Medium (DMEM, Biological Industries, Beit Haemek, Israel) supplemented with 10% fetal calf serum (FCS). For cytotoxicity studies [27], cells were seeded in 96-well plates (1.5 × 10^4^ cells/well) in medium containing ricin (30 ng/mL) and incubated at 37 °C in the presence or absence of the anti-ricin antibody. Six hours later, the medium was removed; the cells were lysed; and the residual intracellular ubiquitin-luciferase fusion protein activity was determined using *D*-luciferin as a substrate and expressed as percent activity determined for untreated cells.

### 4.11. In Vivo Protection Assay

Female outbred ICR mice (Charles River Laboratories, Canterbury, UK) were maintained at 20–22 °C and a relative humidity of 50 ± 10% on a 12-h light/dark cycle, fed with commercial rodent chow (Koffolk Inc., Rancho Santa Fe, CA, USA) and provided with tap water *ad libitum*. Treatment of animals was in accordance with regulations outlined in the U.S. Department of Agriculture (USDA) Animal Welfare Act and the conditions specified in the Guide for Care and Use of Laboratory Animals (National Institute of Health, 2011). Animal studies were approved by the local ethical committee (m-55-2013) on animal experiments.

Anesthetized mice, 27–30 g, were intoxicated by intranasal instillation of 2LD_50_ of ricin (5 µg/kg, 50 µL/mice) that was slowly applied (25 µL/nostril) using a gel-loading tip [19,20,44] and treated with each mAb six hours after intoxication (*n* = 10–22 for each mAb). Antibodies were diluted in PBS to a concentration of 0.5 mg/mL and administered by intravenous injection in a final volume of 200 µL. The mice were monitored for 14 days, and the protection conferred by each antibody was calculated as the percent of surviving mice. Mice infected with ricin without antibody treatment were used as the control. Survival plots were calculated using Prism software (Version 5.01, GraphPad Software Inc., La Jolla, CA, USA, 2007).

## Figures and Tables

**Table 1:** Characteristics of the isolated anti-ricin antibodies.

Antibody	Vaccine ^a^	Binding ^b^	ED_50_ (ng/mL) ^c^	*k*_on_ (1/Ms)	*k*_off_ (1/s)	*K*_D_ (nM)
MH1	subunit	RTA	200 ^d^	9 × 10^4^	<1 × 10^−7^	<0.001
MH2	subunit	RTB	5200	18 × 10^4^	1 × 10^−5^	0.6
MH36	holotoxin	RTA	27,000	11 × 10^4^	<1 × 10^−7^	<0.001
MH49	holotoxin	RTA	1200	7 × 10^4^	<1 × 10^−7^	<0.001
MH67	holotoxin	RTA	52,000	9 × 10^4^	8 × 10^−4^	9.0
MH73	subunit	RTB	11,000	11 × 10^4^	<1 × 10^−7^	<0.001
MH74	subunit	RTA	83,000 ^d^	15 × 10^4^	7 × 10^−3^	45
MH75	subunit	RTB	150 ^d^	35 × 10^4^	<1 × 10^−7^	<0.001
MH76	subunit	RTB	10,500	11 × 10^4^	3 × 10^−4^	3.0
MH77	subunit	RTB	500	17 × 10^4^	1 × 10^−7^	0.001

^a^ The vaccine used to immunize the animal from which the antibody was isolated. ^b^ Binding of each antibody to ricin subunits was determined by enzyme linked immunosorbent assay (ELISA), except for MH73, which was tested by Octet. ^c^ The antibody concentration needed to neutralize 50% (ED_50_) of ricin activity *in vitro.* ^d^ Values taken from our previous study [27] are presented here for comparison.

**Table 2:** Rhesus macaque germline genes most similar to the genes encoding the isolated anti-ricin antibodies.

Antibody	VH			VL	
	V	D	J	V	J
MH1 ^a^	IGHV4.40	IGHD2-4*01	IGHJ1*01	IGLV3.46 ^c^	IGLJ3
MH2 ^a^	IGHV3.15	IGHD5-3*01	IGHJ5-1*01	IGLV3.46 ^c^	IGLJ2
MH36 ^b^	IGHV3.9	IGHD4-2*01	IGHJ4*01	IGLV1.25 ^c^	IGLJ2
MH49 ^b^	IGHV4.11	IGHD4-2*01	IGHJ3*01	IGLV1.27 ^c^	IGLJx1
MH67 ^b^	IGHV4.11	IGHD4-2*01	IGHJ3*01	IGKV1.9 ^d^	IGKJ2
MH73 ^a^	IGHV4.11	IGHD1-2*01	IGHJ5-1*01	IGKV1.52 ^d^	IGKJ2
MH74 ^a^	IGHV5.7	IGHD3-3*01	IGHJ5-2*02	IGLV1.30 ^c^	IGLJ3
MH75 ^a^	IGHV3.15	IGHD5-3*01	IGHJ5-1*01	IGLV3.46 ^c^	IGLJ2
MH76 ^a^	IGHV3.15	IGHD3-1*01	IGHJ4*01	IGLV3.46 ^c^	IGLJ3
MH77 ^a^	IGHV4.40	IGHD4-4*01	IGHJ5-1*01	IGKV1.7 ^d^	IGKJ2

^a^ Originated from the subunit-based vaccine library. ^b^ Originated from the holotoxin-based vaccine library. ^c^ λ isotype. ^d^ *κ* isotype.

## Supplementary Materials

The following are available online at www.mdpi.com/2072-6651/8/3/64/s1. Figure S1: Primers designed for scFv library construction. Figure S2: Multiple alignment of the scFv sequences. Figure S3: Binding of antibody MH73 to ricin chain B.
