# Supplementary material for: Isolation of Anti-Ricin Protective Antibodies Exhibiting High Affinity from Immunized Non-Human Primates
Source: Toxins (Basel). 2016 Mar 3;8(3):64. doi: 10.3390/toxins8030064 (PMC4810209; doi:10.3390/toxins8030064)
Supplement: Supplementary file 1 [file toxins-08-00064-s001.pdf]

Supplementary Materials: Isolation of Anti-Ricin Protective Antibodies Exhibiting High Affinity from Immunized Non-Human Primates

Tal Noy-Porat, Ronit Rosenfeld, Naomi Ariel, Eyal Epstein, Ron Alcalay, Anat Zvi, Chanoch Kronman, Arie Ordentlich and Ohad Mazor

primer	sequence
**VH amplification**	
Lib-H1 F	CTTTCTATGCGGCCCAGCCGG**CCATGG**CCCAGGAGCAGCTGGTGCAGTC
Lib-H2 F	CTTTCTATGCGGCCCAGCCGG**CCATGG**CCCAGGTCCAGCTGGTGSAGWC
Lib-H3 F	CTTTCTATGCGGCCCAGCCGG**CCATGG**CCCAGGTSCAGCTCGAGSAGTC
Lib-H4 F	CTTTCTATGCGGCCCAGCCGG**CCATGG**CCCAGGTGCAGCTGCAGGAGTC
Lib-H5 F	CTTTCTATGCGGCCCAGCCGG**CCATGG**CCCAGCTGCAGCTGCAGSAGTC
Lib-H6 F	CTTTCTATGCGGCCCAGCCGG**CCATGG**CCCAGGTGCAGCTRCTCGAGTS
Lib-H7 F	CTTTCTATGCGGCCCAGCCGG**CCATGG**CCCAGGTSCAGCTGGTGCAGTY
Lib-H8 F	CTTTCTATGCGGCCCAGCCGG**CCATGG**CCCAGGTSACCTTGAAGGAGTC
Lib-H9 F	CTTTCTATGCGGCCCAGCCGG**CCATGG**CCCAGGTCCAGCTGCAGGAAAG
Lib-H10 F	CTTTCTATGCGGCCCAGCCGG**CCATGG**CCGAGGTGCAGYTGGTGGAGWC
Lib-H11 F	CTTTCTATGCGGCCCAGCCGG**CCATGG**CCGAGGTTCAGYTGGTKGAATC
Lib-H12 F	CTTTCTATGCGGCCCAGCCGG**CCATGG**CCGAGGTGCAGCTGGTGSARTC
Lib-H13 F	CTTTCTATGCGGCCCAGCCGG**CCATGG**CCGAGGTGCAGCTGGYRGAGTC
Lib-H14 F	CTTTCTATGCGGCCCAGCCGG**CCATGG**CCGAAGTGCAGYTGGTGGAGTC
Lib-H15 F	CTTTCTATGCGGCCCAGCCGG**CCATGG**CCGAGGTGCAGCTCGAGGAGTC
Lib-H16 F	CTTTCTATGCGGCCCAGCCGG**CCATGG**CCGAGGTGCAGCTGCTCGAGTC
VH rev1	CACCGGATCCTCCTCCTCCT**GCTGAGC**CTGARGAGRCTGTGACC
VH rev2	CACCGGATCCTCCTCCTCCT**GCTGAGC**CTGAGGACACGGCAACC
Vκ amplification	
Lib-K1 F	TCCGGTGGTGGTGGTTCTGGCGGCGGCGGCTCCGATATTGTGATGAYCCAGAC
Lib-K2 F	TCCGGTGGTGGTGGTTCTGGCGGCGGCGGCTCCGATACTGTGATGACCCAGAC
Lib-K3 F	TCCGGTGGTGGTGGTTCTGGCGGCGGCGGCTCCGATATYGAGCTCACBCAGTC
Lib-K4 F	TCCGGTGGTGGTGGTTCTGGCGGCGGCGGCTCCGATGTTGYRATGACTCAGTC
Lib-K5 F	TCCGGTGGTGGTGGTTCTGGCGGCGGCGGCTCCGACATTCAGMTGWCCCAGTC
Lib-K6 F	TCCGGTGGTGGTGGTTCTGGCGGCGGCGGCTCCGACGTTCAGATGACCCAGTC
Lib-K7 F	TCCGGTGGTGGTGGTTCTGGCGGCGGCGGCTCCGACATCCAGATGACCCAGTC
Lib-K8 F	TCCGGTGGTGGTGGTTCTGGCGGCGGCGGCTCCGAGCTCCWGATGACMCAGTC
Lib-K9 F	TCCGGTGGTGGTGGTTCTGGCGGCGGCGGCTCCGAAATWGTRATGACGCAGTC
Lib-K10 F	TCCGGTGGTGGTGGTTCTGGCGGCGGCGGCTCCGAAATCGAGCTCACRCAGTC
Lib-K11 F	TCCGGTGGTGGTGGTTCTGGCGGCGGCGGCTCCCAAGTTATATTGACTCAGTC
Lib-K12 F	TCCGGTGGTGGTGGTTCTGGCGGCGGCGGCTCCGACATCGAGCTCACCCAGTC
Lib-K13 F	TCCGGTGGTGGTGGTTCTGGCGGCGGCGGCTCC GAGCTCGTGTTGACACAGTC
Lib-K1 Rev	GATACCGGTGTATTTGCGCCACCT**GCGGCCGC**YTTGAKATCCAGTTTGGTCCCGGG
Lib-K2 Rev	GATACCGGTGTATTTGCGCCACCT**GCGGCCGC**TTTGAYCTCCACCYTGGTCCCTCC
Lib-K3 Rev	GATACCGGTGTATTTGCGCCACCT**GCGGCCGC**TTTGATSTCCACTTTGGTCCCCTG
Lib-K4 Rev	GATACCGGTGTATTTGCGCCACCT**GCGGCCGC**TYTGATTTCCACCYTGGTCCCTTG
Lib-K5 Rev	GATACCGGTGTATTTGCGCCACCT**GCGGCCGC**CTTGATGTCCACCTTGGTCCCGTG
Lib-K6 Rev	GATACCGGTGTATTTGCGCCACCT**GCGGCCGC**TTTTAGTACCACCTTGGTCCCTTG
**V**λ amplification	
Lib-L1 F	TCCGGTGGTGGTGGTTCTGGCGGCGGCGGCTCCCAGCCAGKGCTGACTCAGCC
Lib-L2 F	TCCGGTGGTGGTGGTTCTGGCGGCGGCGGCTCCCAGCCTGKGCTGACTCAGYC
Lib-L3 F	TCCGGTGGTGGTGGTTCTGGCGGCGGCGGCTCCCAGTCTGTGYTGACKCAGCC
Lib-L4 F	TCCGGTGGTGGTGGTTCTGGCGGCGGCGGCTCCCAGTCTGCCCTGACTCAGCC
Lib-L5 F	TCCGGTGGTGGTGGTTCTGGCGGCGGCGGCTCCCAGTCTGCCCCGAYTCAGYC
Lib-L6 F	TCCGGTGGTGGTGGTTCTGGCGGCGGCGGCTCCCAGGCTGCCCYGACTCAGYC
Lib-L7 F	TCCGGTGGTGGTGGTTCTGGCGGCGGCGGCTCCCAGGCAGGGCTGACTCAGCC
Lib-L8 F	TCCGGTGGTGGTGGTTCTGGCGGCGGCGGCTCCCAGACTGTGGTGACCCAGGA
Lib-L9 F	TCCGGTGGTGGTGGTTCTGGCGGCGGCGGCTCCAAGCCTATGCTGACTCAGCC
Lib-L10 F	TCCGGTGGTGGTGGTTCTGGCGGCGGCGGCTCCTCTTCTGRGCTGACTCAGGA
Lib-L11 F	TCCGGTGGTGGTGGTTCTGGCGGCGGCGGCTCCTCCTATGAGCTGACWCAGCC
Lib-L12 F	TCCGGTGGTGGTGGTTCTGGCGGCGGCGGCTCC CAGSCTGTGCTGACTCAGCC
Lib-L13 F	TCCGGTGGTGGTGGTTCTGGCGGCGGCGGCTCC CWGCCTGTGCTGACTCARYC
Lib-L1 Rev	CCGGTGTATTTGCGCCACCT**GCGGCCGC**TAGRACGGTSAGCCGGGTC
Lib-L2 Rev	CCGGTGTATTTGCGCCACCT**GCGGCCGC**GAGGAYGGTCAAYTTGGTG
**Linker addition primer for**	
Forward 2	TCACAGTCTCCTCAG**GCTCAGC**AGGAGGAGGAGGATCCGGTGGTGGTGGTTCTGG
**Primers for scFv assembly**	
ASS1 For	CTTTCTATGCGGCCCAGC
ASS1 Rev	GATACCGGTGTATTTGCG
	**Primers for colony PCR and sequencing**
TAB-RI For	CCATGATTACGCCAAGCTTTGGAGCC
CBD-AS Rev	GAATTCAACCTTCAAATTGCC

Figure S1. Primers designed for scFv library construction.

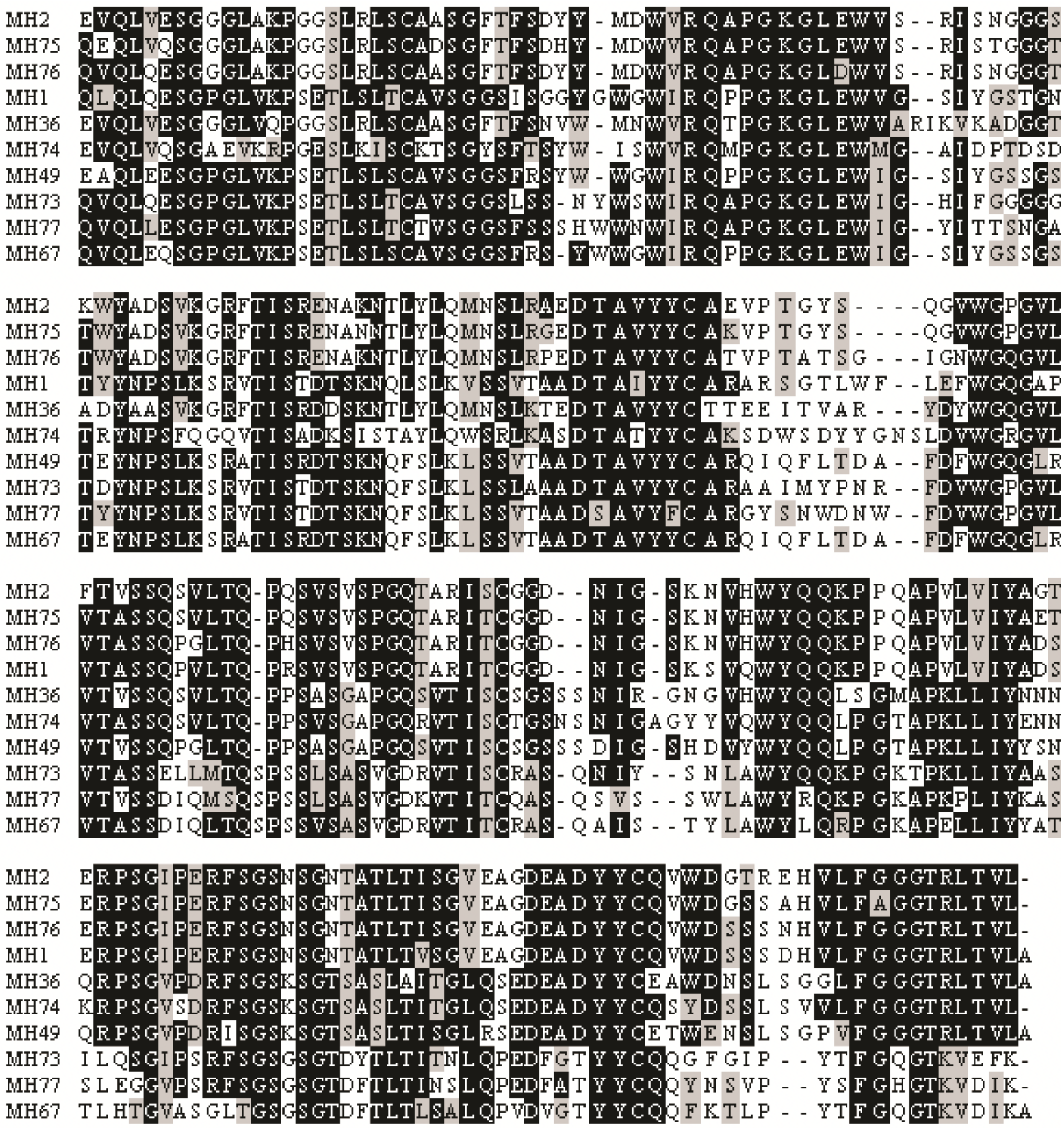

Figure S2. Multiple alignment of the scFv sequences.

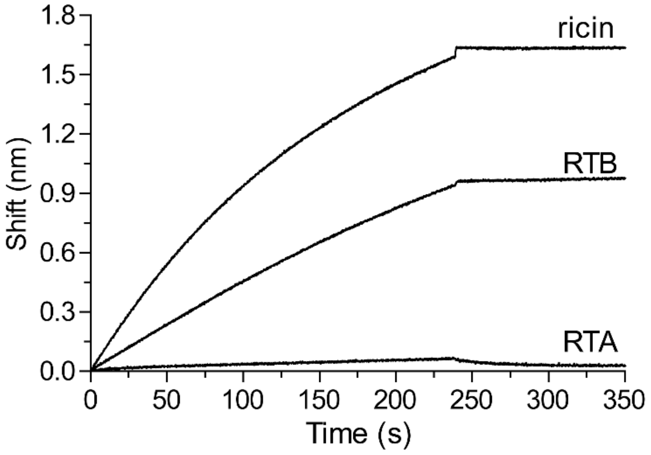

Figure S3. Binding of antibody MH73 to ricin chain B.
